# Role of Melatonin in Regulating Rat Skeletal Muscle Tissue Inflammation and Damage Following Carbon Tetrachloride Intoxication

**DOI:** 10.3390/ijms26041718

**Published:** 2025-02-17

**Authors:** Vladimir Milan Antić, Milorad Antic, Nenad Stojiljkovic, Nemanja Stanković, Miljana Pavlović, Dušan Sokolović

**Affiliations:** 1 Faculty of Sports and Physical Education, University of Niš, 18000 Niš, Serbia; vlada.antic@hotmail.com (V.M.A.); snesadif@yahoo.com (N.S.); nemanjastankovic84@hotmail.com (N.S.); 2 Department of Anatomy, Faculty of Medicine, University of Niš, 18000 Niš, Serbia; antic.miki87@gmail.com (M.A.); pavlovic_miljana@yahoo.com (M.P.); 3 Department of Biochemistry, Faculty of Medicine, University of Niš, 18000 Niš, Serbia

**Keywords:** skeletal muscles, CCl_4_, inflammation, CD45, nitric oxide

## Abstract

Carbon tetrachloride (CCl_4_) is a toxic compound that causes severe oxidative stress and inflammation in skeletal muscles, resulting in structural damage, mitochondrial dysfunction, and impaired contractile function. While CD45 and melatonin (MLT) are implicated in immune modulation and antioxidative defense, their precise roles in mitigating CCl_4_-induced muscle damage remain incompletely understood, warranting further investigation. This study used 24 Wistar rats divided into four groups to evaluate the effects of MLT on CCl_4_-induced muscle inflammation. The first group was used as a control group, the second received only MLT (50 mg/kg), and the third group received CCl_4_, while the fourth group received MLT (50 mg/kg) and CCl_4_. Muscle tissues, obtained 24 h after the commencement of the experiment, were analyzed using biochemical assays for inflammatory markers, histological staining, and immunohistochemistry to assess structural and cellular changes. CCl_4_ exposure significantly increased NF-κB activity, nitric oxide levels, iNOS expression, and CD45-positive immune cell infiltration in skeletal muscles, indicating heightened inflammation and oxidative stress. Pretreatment with MLT markedly reduced these inflammatory markers, restoring damaged tissue and diminishing immune cell infiltration. Histological analyses confirmed reduced inflammatory cell presence and tissue damage in MLT-treated animals, highlighting its protective effects. Melatonin demonstrates significant protective effects against CCl_4_-induced skeletal muscle damage by reducing inflammation, oxidative stress, and immune cell infiltration, highlighting its potential as a therapeutic agent.

## 1. Introduction

Carbon tetrachloride (CCl_4_) is a toxic chemical compound that, when introduced into the body, can lead to severe damage in various organs, including skeletal muscles [1,2]. It is used in laboratory settings (solvent), as a synthetic industrial chemical, and sometimes as a pesticide. The harmful effects of CCl_4_ on skeletal muscles are primarily attributed to the production of free radicals and oxidative stress, initiating lipid peroxidation, cell membrane damage, and disruption of cellular integrity. Additionally, CCl_4_-induced oxidative stress can lead to mitochondrial dysfunction, impaired energy production, and activation of apoptotic pathways in skeletal muscle cells [3,4]. As a consequence, the skeletal muscle tissue experiences inflammation, necrosis, and degeneration, ultimately leading to muscle weakness, loss of contractile function, and even permanent structural damage. The severity of CCl_4_-induced skeletal muscle damage depends on factors such as the duration and concentration of exposure [2,5]. Preventing exposure to CCl_4_ and early intervention with antioxidants or anti-inflammatory agents may offer some protection against its deleterious effects on skeletal muscles. However, it is crucial to handle and use this chemical with extreme caution to avoid potential harm to both human health and the environment. When muscle tissue is exposed to CCl_4_ during a more extended period, an elevation in dystrophin as a response to TGF-β1 occurs [6].

CD45, also known as leukocyte common antigen (LCA), is a transmembrane protein tyrosine phosphatase that plays a crucial role in the immune system [7]. While it is primarily associated with immune cells, such as T cells, B cells, and macrophages, CD45 has also been found to have some functions in skeletal muscles. In skeletal muscles, CD45 is expressed on muscle-resident immune cells, such as macrophages and regulatory T cells, and has been implicated in modulating the immune response during muscle injury and regeneration [8]. While the role of CD45 in skeletal muscles is not as well understood as its functions in the immune system, emerging research suggests that this protein may play a significant role in coordinating immune responses and tissue repair processes during muscle injury and regeneration. Thus, further investigations are needed to fully elucidate the precise mechanisms through which CD45 influences skeletal muscle physiology and pathology.

Melatonin (MLT), a methoxyindole, is a potent natural antioxidant, immunostimulant, protein synthesis regulator, and anticancer agent primarily synthesized by pineal gland cells during nighttime [9]. It was initially isolated from bovine pineal tissue; now, MLT is known to be synthesized by many cells and organs. MLT’s main function is to convey information about the daily light-dark cycle to the body, thus regulating circadian rhythms, core temperature, sleep–wake cycles, immune function, antioxidative defenses, hemostasis, and glucose levels [10]. MLT directly scavenges oxygen- and nitrogen-based free radicals, enhances antioxidant enzyme synthesis, and boosts the production of non-enzymatic antioxidants [10]. This makes MLT a promising therapeutic agent for different disorders, given its low toxicity and specific receptor targeting. Studies on MLT’s role in preventing different tissue damage induced by CCl_4_ and its effects on inflammation have been studied. However, there is no complete understanding of its effects.

The present study aims to evaluate the effect of CCl_4_ application on inflammatory changes in rat muscle tissue and the potential protective effects of MLT in this process. The process of inflammation would be followed through a number of biochemical parameters in the muscle tissue and using pathohistological/immunohistochemical methods to corroborate the biochemical findings.

## 2. Results

**Figure 1:**
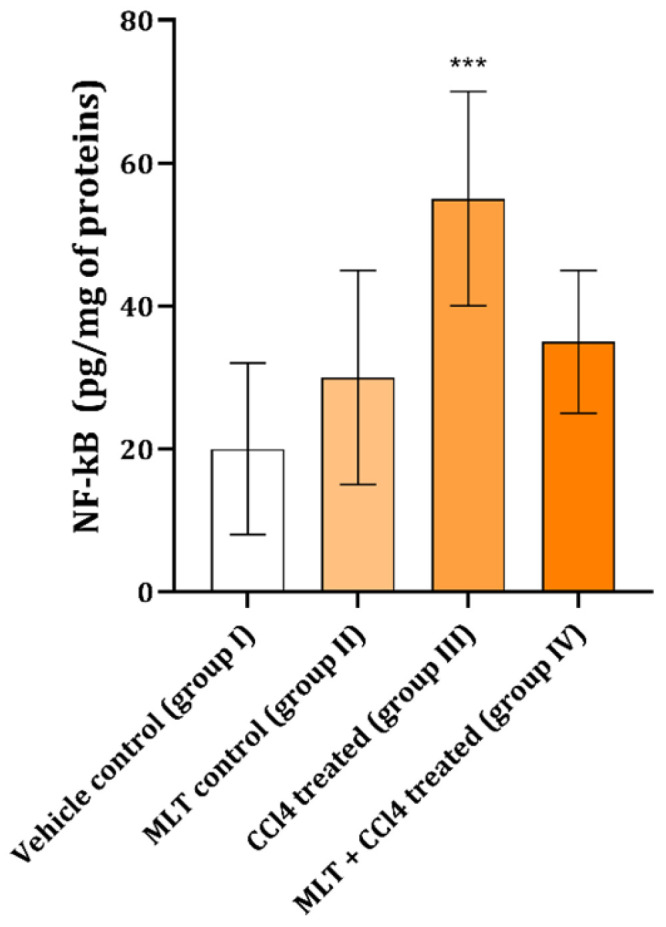
NF-kB content in animals belonging to different groups. The data are presented as mean ± SD (n = 6). The comparison was performed using one-way ANOVA followed by Tukey’s post hoc test. *** *p* < 0.05 vs. control.

Application of CCl_4_ to rats produced a significant increase in NF-kB (Figure 1) compared with control and MLT-treated animals. The animals exposed to MLT prior to CCl_4_ had the NF-kB content not different from the experimental group (Figure 1).

**Figure 2:**
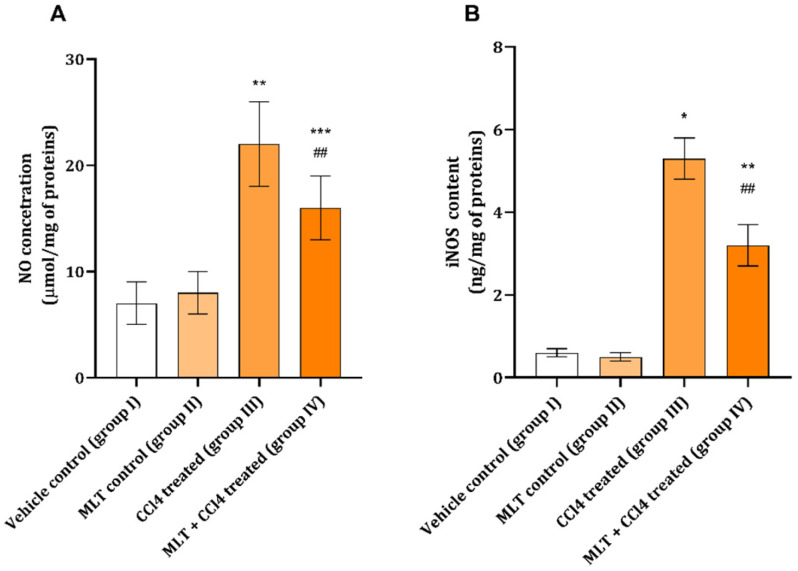
Effect of MLT on CCl_4_ muscle tissue (**A**) nitric oxide (μmol/mg of proteins) and (**B**) iNOS (ng/mg of proteins) concentrations. The data are presented as mean ± SD (n = 6). The comparison was carried out using one-way ANOVA followed by Tuckey’s post hoc test, * *p* < 0.001, ** *p* < 0.01, *** *p* < 0.05 vs. control, ^##^ *p* < 0.01 vs. CCl_4_-treated animals.

During the experiment, the amount of NO and iNOS levels were significantly increased in animals exposed to CCl_4_ compared with the control (Figure 2A,B). When animals were pretreated with MLT prior to CCl_4_, the levels of the two investigated parameters were found to be lower than in the CCl_4_ treated animals, but still significantly different from the control (Figure 2A,B).

**Figure 3:**
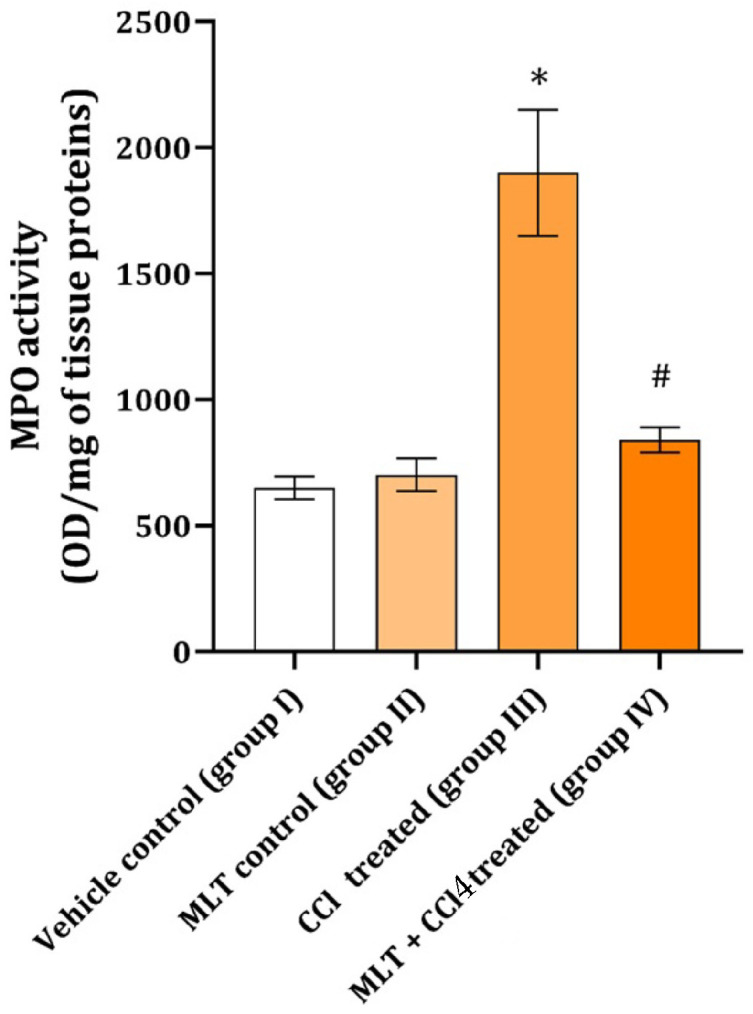
Effect of MLT on CCl_4_ muscle tissue MPO activity. The data are presented as mean ± SD (n = 6). The comparison was carried out using one-way ANOVA followed by Tuckey’s post hoc test, * *p* < 0.001 vs. control, ^#^ *p* < 0.001 vs. CCl_4_-treated animals.

Muscle tissue MPO activity was found to be statistically significantly increased in animals treated with CCl_4_ only (Figure 3). The activity of this enzyme in the group that received MLT and CCl_4_ was significantly different from that of CCl_4_ and closer to that of the control group (Figure 3).

**Figure 4:**
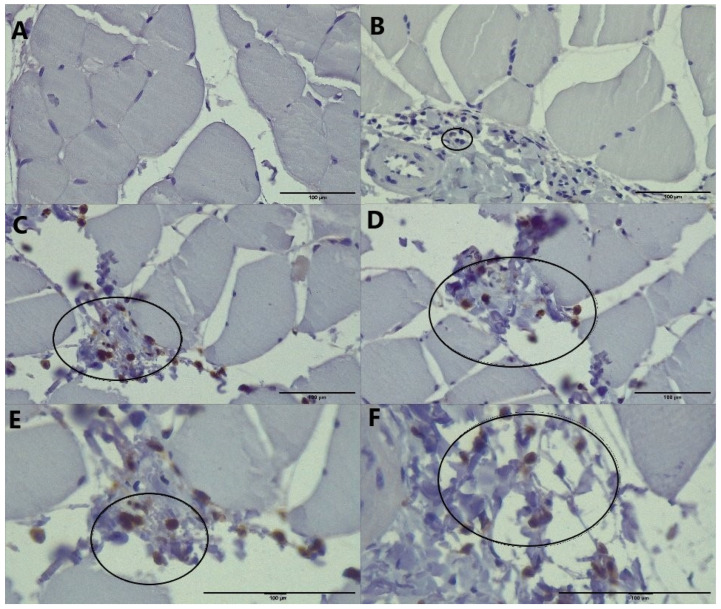
Skeletal muscle tissue of rats belonging to control (**A**), MLT (**B**), CCl_4_ (**C**,**E**), and MLT + CCl_4_ (**D**,**F**) group stained for CD45 expression in inflammatory cells—circled (×400 and ×630 for **E**,**F**).

Muscle tissue stained for CD45 expression revealed no positivity of these cells in control and very rare MLT-treated animals (Figure 4A,B). Only the number of positive cells was pronounced in the group that received CCl4 (Figure 4C,E), while in those that received MLT and CCl4, CD45 positivity occurred occasionally (Figure 4D,F).

Inflammatory cell infiltration was found to be increased in the groups of animals exposed to CCl_4_, while in the group that received MLT prior to CCl_4_, this infiltration was much less (Table 1). The number of CD45 cells was found to be increased in all animals receiving CCl_4_, while in the group that received MLT with CCl_4_, this increase was milder compared with the group receiving only CCl_4_ (Table 1).

## 3. Discussion

Administration of CCl_4_ to rats has been established as a model of skeletal tissue damage, which is followed by serum and tissue biochemical changes indicative of cell damage [2,5]. Also, signs of inflammation such as inflammatory cell infiltration [2] and an increased number of degranulated mastocytes [1] following CCl_4_ application. After muscle damage, immune cells infiltrate the injured area to clear cellular debris and promote tissue repair. This process is partially mediated by CD45-expressing immune cells, which interact with other immune cells and muscle cells through different signaling mechanisms [8].

Exposure of animals to CCl_4_ leads to the alteration in NF-kB expression and activity [11], which finally results in cell production of different cytokines (e.g., TNF-α, IL-1β, and COX-2) [12]. Moderate muscle damage through physical exercise and/or electrical stimulation could cause NF-kB activation, further regulating the NOS pathway and NO production [13]. One of the NF-kB activators is liberated MPO and its products (degraded H_2_O_2_); however, this is controversial. It is speculated that MPO activates CD11b receptors, while HOCl activates NF-kB translocation and induction of chemokine production [14]. The activity of MLT in preventing NF-kB could be through the potential reduction (scavenging) of free radicals [4] and through the modulation of MPO activity [3]. Thus, overall, it would impact the NF-kB signaling and the production of cytokines.

One of the signaling mechanisms associated with inflammation, but with tissue regeneration as well, is the nitric oxide signaling pathway. This pathway can be connected with both an increase and decrease in muscle contractile function depending on multiple factors, with the main ones being a source of NO, intensity of reaction, and duration of signaling [15]. In the present study, only a moderate increase in NO concentrations (Figure 2A), followed by an increase in iNOS (Figure 2B), has been detected in animals exposed to a damaging agent, CCl_4_. The cells secreting these minute amounts of NO should be part of CD45-positive infiltrating cells in the muscle tissue (Figure 4C). Melatonin application prior to CCl4 caused a diminution in NO production and iNOS expression, which has been proven in other models of tissue damage induced by CCl_4_ [3,4]. Nitric oxide is potentially increased through the activated NF-kB pathway, which has already been described.

Interestingly, since the detected levels of NO are relatively low, they potentially do not negatively decrease NF-kB as one might expect under extensive inflammation [16]. Other sources of NO could also be traced to mast cells, which can liberate their granules when activated by CCl_4_ in muscle tissue [1]. Finally, a decrease is also visible through a much lower number of CD45 infiltrating cells observed in the group of animals treated with MLT and CCl_4_ (Figure 4D).

It has been shown that MPO is responsible for around 50% of skeletal muscle cell damage and cell membrane lysis in animals during different experimental conditions involving mechanical muscle damage [17]. Since MPO is a macrophage and/or neutrophile-borne enzyme [3], its association with CD45 expressing inflammatory cells can be brought in connection. Thus, an increased number of cells expressing CD45 could be connected with increased MPO activity in the muscles of rats from the CCl_4_ group. On the other hand, a reduction in CD45 expressing inflammatory cells clearly follows the pattern of MPO decrease detected in a group of animals receiving both CCl_4_ and MLT (Figure 3). The activity of the MPO CCl_4_ group might correspond to M1 inflammatory macrophages and neutrophils, while their diminution in the CCl_4_ and MLT groups could be either from lesser infiltrate or from the faster transformation into M2 [18] by the applied MLT. Thus, a fine balance between inflammation and its prevention is needed to prevent tissue damage and establish and activate adequate repair mechanisms.

Recent studies suggest that CD45-positive macrophages, a source of both NO and MPO, can influence muscle regeneration by promoting the differentiation of muscle stem cells (myoblasts) into mature muscle fibers (myofibers). These macrophages release factors that stimulate myoblast proliferation and fusion, ultimately aiding in the restoration of damaged muscle tissue [19]. Moreover, CD45 has been shown to regulate inflammatory signaling pathways in muscle cells, helping to control excessive inflammation and prevent further tissue damage during injury. It is believed that CD45 acts as a modulator of immune responses in skeletal muscles, contributing to the delicate balance between inflammation and tissue repair [20]. After the muscle healing process is finished, the infiltrated cells will gradually disappear [20]. These roles of CD45-positive macrophages are partially in agreement with the results, which point to the involvement of these cells in the processes occurring in muscle tissue in animals treated with MLT and CCl_4_.

The effects of MLT could also be mediated via specific G protein-coupled melatonin receptors (MT1 and MT2) expressed on immune cells. Their activation inhibits leukotriene production and stimulates IL-2 and IL-6, thus altering immune response [21]. Also, MLT could alter oxidative damage via activation of specific receptors such as aryl hydrocarbon receptor (AhR) and peroxisome proliferator-activated receptor (PPAR)γ expressed on different cells, including immune system cells [22]. The alteration in the function of the immune system is one of the mechanisms by which MLT might prevent skeletal muscle damage, as observed in the present study.

The role of MLT in the healing process of muscle damage induced by CCl_4_ might be associated with either their role in preventing cell damage through previously described mechanisms [2] or through mechanisms associated with diminution of cell infiltration or by change in their profile. Namely, MLT could modulate and enhance the maturation of CD8+ cytotoxic lymphocytes [23], which could eventually modulate the damaged muscle. Also, MLT is known to alter cell immunological profile by decreasing the expression of various surface molecules on mesenchymal stem cells, including CD45 [24]. Since the observed results only point to a slight diminution in both inflammatory infiltration and CD45 expression in the group of animals treated with MLT and CCl_4_, it is hard to determine the precise role of this neurohormone.

This study has some limitations, which are reflected in the preliminary approach to the topic of changes in the skeletal muscle inflammatory response during exposure to CCl_4_ and MLT. Although preliminary, this study approached the issue of tissue inflammation via several possible ways using standard routine biochemical and immunohistochemical methods for the quantification of inflammation-related parameters. Further studies should be designed in order to evaluate muscle tissue molecular events happening under the described experimental conditions.

## 4. Materials and Methods

### 4.1. Drugs and Chemicals

All chemicals and reagents used in this experiment were purchased from Sigma-Aldrich (St. Louis, MO, USA) and Carl Roth (Karlsruhe, Germany) and were of the highest commercial and analytical grade available. Melatonin (MLT, >95%) was dissolved in absolute ethanol and further diluted in a sterile saline solution (0.9% NaCl) to a final concentration of <0.1% ethanol before intraperitoneal administration at a dose of 50 mg/kg. The dose, treatment regimen, and route of application were based on previous publications.

### 4.2. Animals and Housing

A total of 24 male Wistar rats (250–300 g) were divided into groups of 6 animals (Institute of Biomedical Sciences, Faculty of Medicine, University of Niš, Niš, Serbia). Animals were housed under standard laboratory conditions, with food and water available ad libitum. All experimental procedures were carried out according to the Declaration of Helsinki and Europe Community Guidelines for the Ethical Use of Laboratory Animals (2010 EU Directive; 2010/63/EU) and were approved by the local Ethics committee.

### 4.3. Experimental Design

Muscle tissue inflammation was induced by injecting a single dose of CCl4 (50%, *v*/*v*) as established and described in previous studies [1,2]. The groups were treated as follows: Group I (vehicle control) received vehicle (0.85% NaCl saline solution containing 8% ethyl alcohol) 24 h before the end of the experiment; Group II (MLT control) received MLT (50 mg/kg) [1,2,3,4]; Group III (CCl_4_ control) received a single dose of CCl_4_ (1 mL/kg); and Group IV (MLT + CCl_4_) received MLT (50 mg/kg) one hour before the CCl_4_ dose.

All animals were sacrificed 24 h after the commencement of the experiment by an overdose of ketamine (Ketamidor, 10%; Richter Pharma AG, Wels, Austria). Skeletal muscle tissues (biceps muscle) were collected for histological analyses, while the opposite muscle was snap-frozen and kept at −80 °C until further biochemical analysis.

### 4.4. Tissue Collection and Preparation for Biochemical Assays

Isolated biceps muscle tissue was homogenized in phosphate-buffered saline (PBS), and 10% tissue homogenate (*w*/*v*) was made using a mechanical homogenizer. Afterward, homogenates were centrifuged at 10,000 rpm for 15 min at 4 °C, and clear supernatants were used for the determination of biochemical parameters. Homogenate protein content was determined using Lowry’s method, and the amount was calculated based on the albumin standard curve [25].

### 4.5. Biochemical Parameters Determination

#### 4.5.1. Myeloperoxidase Activity Determination

Muscle tissue MPO activity was determined using a standard protocol previously described [3]. Briefly, tissue homogenate was incubated under acidic conditions with ο-phenylenediamine, which was activated with H_2_O_2_. The reaction was stopped by adding 2M H_2_SO_4_, and optical densities (OD) were determined at 540 nm using a microplate reader. The results are expressed as OD/mg of proteins.

#### 4.5.2. Nitric Oxide Determination

Following the deproteinization of tissue samples, the concentration of nitrate and nitrite was determined using the Griess colorimetric method [26]. As the Griess reagent selectively reacts with nitrites, cadmium was employed to reduce nitrate to nitrites. Subsequently, after the addition of the Griess reagent (comprising 1.5% sulfanilamide in 1 M HCl plus 0.15% N-(1-naphthyl)), absorbance readings were taken at 543 nm.

#### 4.5.3. Inducible Nitric Oxide Synthase Determination

The determination of iNOS in rat muscle tissue was performed using CUSBIO (CSB-E08325r) sandwich enzyme immunoassay kit following the manufacturer’s instructions. The obtained values are expressed as IU/mg of tissue proteins.

#### 4.5.4. Tissue NF-Kappa-B-Activating Protein

Commercial kits for determining the NF-κB level (NF-kappa-B-activating protein ELISA Kit, Wuhan Fine Biotech, Wuhan, China; ER0510) were used for protein level determination. Determination was conducted according to the manufacturer’s instructions. The concentrations of the determined parameter were presented as pg/mg of proteins.

#### 4.5.5. Tissue Processing, Histochemical and Immunohistochemical Staining

The tissue samples designated for histopathological analysis were fixed in a 10% (*w*/*v*) buffered formaldehyde solution. Following fixation, the tissues were dehydrated using ethanol solutions of increasing concentrations (50–100%, *v*/*v*), embedded in paraffin, sectioned into slices 4–5 μm thick, and further stained. Standard hematoxylin–eosin (H&E) staining protocol was applied for staining tissue sections [27]. In the case of immunohistochemical staining, a primary rabbit polyclonal CD45 antibody (CD45, Abcam) was used. Antigen retrieval was performed in citrate buffer, and endogenous peroxidase blockage was performed using 3% hydrogen peroxide. The incubation with the primary antibody was performed overnight in a moist chamber. Afterward, the sections were incubated with appropriate secondary antibody with avidin–biotin immunoperoxidase complex detection system. The visualization was performed using diaminobenzidine and counterstained with Mayer’s hematoxylin.

#### 4.5.6. Microscopic Tissue Analysis

Stained tissue sections were further examined under an Olympus BH2 light microscope (Olympus America Inc., Miami, FL, USA), and digital photographs were acquired using the imaging system (Olympus cellSens platform standard, Olympus Corporation, Tokyo, Japan). Tissue damage changes were assessed on H&E-stained sections using an ×40 magnification lens. The number of inflammatory cells was examined on 5–10 randomly selected high-power fields (HPF) (×40) and scored as absent (0), mild (1), moderate (2), or severe (3), following the criteria described by Rizo-Roca [28]. The number of CD45 antigens was also counted on 5–10 randomly selected HPFs.

#### 4.5.7. Statistical Analysis

Data presented as mean ± SD were compared using one-way analysis of variance (ANOVA), followed by Tukey’s post hoc test for multiple comparisons (GraphPad Prism, ver. 5.03; San Diego, CA, USA). Probability values (*p*) less than 0.05 were considered to be statistically significant.

## 5. Conclusions

This study highlights the severe oxidative stress, inflammation, and tissue damage in skeletal muscles caused by carbon tetrachloride (CCl_4_), primarily through NF-κB activation, nitric oxide production, and immune cell infiltration. Pretreatment with melatonin (MLT) significantly mitigated these effects, reducing inflammation and preserving muscle integrity via its antioxidant and immune-modulating properties. Since the observed results only point to a diminution in both inflammatory infiltration and CD45 expression, as well as a decrease in biochemical inflammatory parameters in the group of animals treated with MLT and CCl_4_, it is hard to determine the precise role of this neurohormone apart from its protective action. Thus, despite these promising results, the precise mechanisms of MLT’s action, especially its influence on immune cell profiles, remain partially unclear. However, the findings emphasize MLT’s therapeutic potential in preventing CCl_4_-induced muscle damage and warrant further investigation into its applications.

## Figures and Tables

**Table 1:** Pathological scores obtained for muscle tissue from different experimental animal groups.

Traced Parameter	Vehicle Control (Group I)	MLT Control (Group II)	CCl_4_ Treated (Group III)	MLT + CCl_4_ Treated (Group IV)
Score of inflammatory cell infiltration	0.1	0.1	1.55	0.6
CD45 cell infiltration (number per HPF)	0	0	7 ± 1	3 ± 1

## Data Availability

Data is available upon reasonable request.

## Abbreviations

CCl_4_	Carbon tetrachloride
CD11b	Cluster of differentiation 11b
CD45	Cluster of differentiation 45
COX-2	Cyclooxigenase-2
H&E	Hematoxylin–eosin
HPF	High-power field
IL-1β	Interleukin 1 beta
iNOS	Inducible nitric oxide synthase
LCA	Leukocyte common antigen
MLT	Melatonin
MPO	Myeloperoxidase
NF-kB	Nuclear factor kappa-light-chain-enhancer of activated B cells
NO	Nitric oxide
OD	Optical densities
PBS	Phosphate-buffered saline
TNF-α	Tumor necrosis factor alfa
TGF-β1	Transforming growth factor beta 1
